# Supplementary material for: Invasive Traits of Symphyotrichum squamatum and S. ciliatum: Insights from Distribution Modeling, Reproductive Success, and Morpho-Structural Analysis
Source: Biology (Basel). 2025 Jan 9;14(1):47. doi: 10.3390/biology14010047 (PMC11762991; doi:10.3390/biology14010047)
Supplement: Supplementary file 1 [file biology-14-00047-s001.zip › Table S1_revised_changes_accepted.pdf]

Table S1 Environmental context of *Symphyotrichum squamatum* and *S. ciliatum* populations from Romania. The table presents environmental variables related to the species' distribution, including soil characteristics, human impact, and topographic factors. The values for the parameters' range/coverage were derived in ArcMap v.10.4 using the *Extract values to points* function, based on various open-source datasets described in the Material and Methods section (Subsection 2.3. Environmental context of populations from Romania)

	Description	Units	*Symphyotrichum squamatum* Range/coverage	*Symphyotrichum ciliatum* Range/coverage
**SOIL CHARACTERISTICS**				
Soil Class	Additional classes (urban)	Categorical	57.6%	0%
	Additional classes (water bodies)		3.8%	1.9%
	Argillic soils		7.6%	7.2%
	Cambisols		0%	5.9%
	Halomorphic soils		0%	1.3%
	Hydromorphic soils		0%	5.2%
	Mollisols		7.6%	53.6%
	Unweathered truncated soils or dissected soils		23%	24.5%
Soil Texture	Clay	Categorical	0%	19.6%
	Clayey		0%	1.9%
	Clayey clay		0%	5.2%
	Clayey loam		0%	3.9%
	Clayey-silt		15.3%	16.9%
	Heterogeneous texture		11.5%	20.9%
	Peat		0%	2.6%
	Sandy		0%	1.9%
	Sandy clay		0%	9.8%
	Sandy clay loam		0%	1.9%
	Sandy clayey loam		0%	4.5%
	Sandy-loam		11.5%	8.4%
	Water bodies (lakes, ponds, marshes)		61.5%	1.9%
**HUMAN IMPACT**				
Human Impact Index (HII)	Ranges from 1 to 100, estimating the relative anthropogenic impact	#	30; 58	10; 60
**TOPOGRAPHIC FACTORS**				
Distance to watercourses	Calculated path distance to watercourses using DEM as a surface raster	m	0; 1170.9	0; 4678.4
Land Use type	Arable land	Categorical	3.8%	44.1%
	Artificial areas - settlements		53.8%	21.7%
	Forests, shrublands		0%	13.9%
	Gravel, sand, dump sites		0%	0.7%
	Lakes		0%	2.3%
	Rivers		11.5%	3.1%
	Roads and infrastructure		26.9%	0.7%
	Vineyards		0%	5.4%

Wetlands and swamps	3.8%	7.7%
