# Supplementary material for: Invasive Traits of Symphyotrichum squamatum and S. ciliatum: Insights from Distribution Modeling, Reproductive Success, and Morpho-Structural Analysis
Source: Biology (Basel). 2025 Jan 9;14(1):47. doi: 10.3390/biology14010047 (PMC11762991; doi:10.3390/biology14010047)
Supplement: Supplementary file 1 [file biology-14-00047-s001.zip › Table S2_revised_changes_accepted.pdf]

Table S2 Anatomical parameters of the vegetative body of *Symphyotrichum squamatum* and *S. ciliatum*. Anatomical measurements for both species were derived from cross-sections taken at standardized levels: the median third of the primary root, the lower and upper thirds of the main stem, and the median third of the lamina. All anatomical features were measured from micro-photographs obtained following double staining with Iodine Green and Carmin Alum, using the software ImageJ. The values represent the mean ± standard deviation for each anatomical parameter.

Organ	Parameter	Measurements / sizes	
		Symphyotrichum squamatum	*Symphyotrichum ciliatum*
Root	Root area (mm^2)	0.64 ± 0.09	0.27 ± 0.23
	Xylem area (mm^2)	0.07 ± 0.04	0.1 ± 0.1
	Aerenchyma area (mm^2)	0.37 ± 0.11	0.08 ± 0.05
Stem	Maximum xylem thickness at the stem base (µm)	809.8	2456
	Aerenchyma thickness at the stem base (µm)	544.8	676.7
	Maximum xylem thickness at the stem apex (µm)	274.2	617.3
	Aerenchyma thickness (in the coastal area) at the stem apex (µm)	306.6	143.2
Lamina	Conducting bundle - median nervure area (length/width) (µm)	157.7/143.8	81.8/48.3
	Palisade tissue height (µm)	152.9	176.1
	Spongy tissue height (µm)	48.4	42
	Palisade cells length/width (µm)	31.2/12.5	37/15.9
	Spongy cells length/width (µm)	11.9/15.2	31.4/32.8
