# Supplementary figures and images for: Invasive Traits of Symphyotrichum squamatum and S. ciliatum: Insights from Distribution Modeling, Reproductive Success, and Morpho-Structural Analysis
Source: Biology (Basel). 2025 Jan 9;14(1):47. doi: 10.3390/biology14010047 (PMC11762991; doi:10.3390/biology14010047)

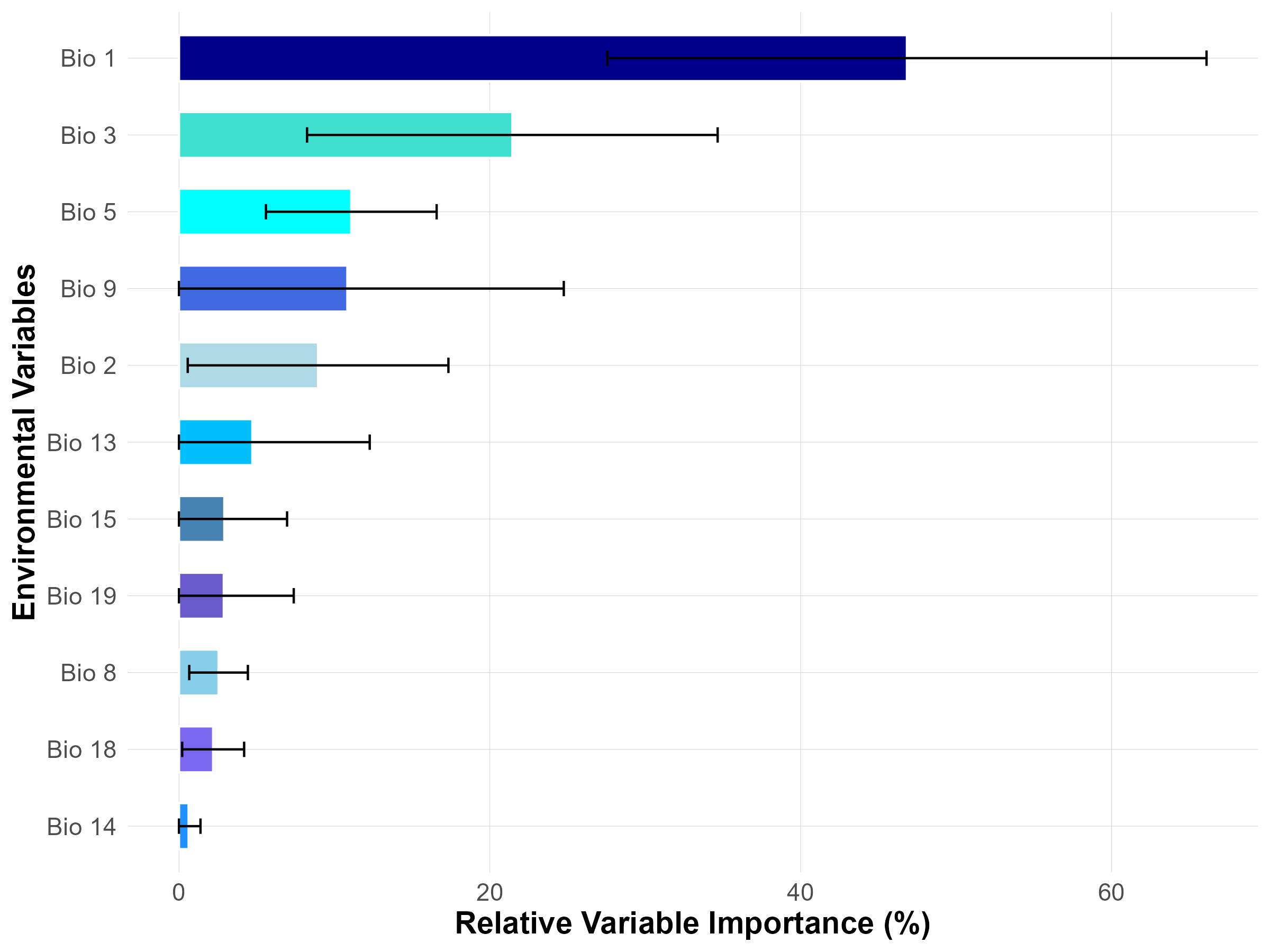

Supplement: Supplementary file 1 [file biology-14-00047-s001.zip › Figure S1.tif]

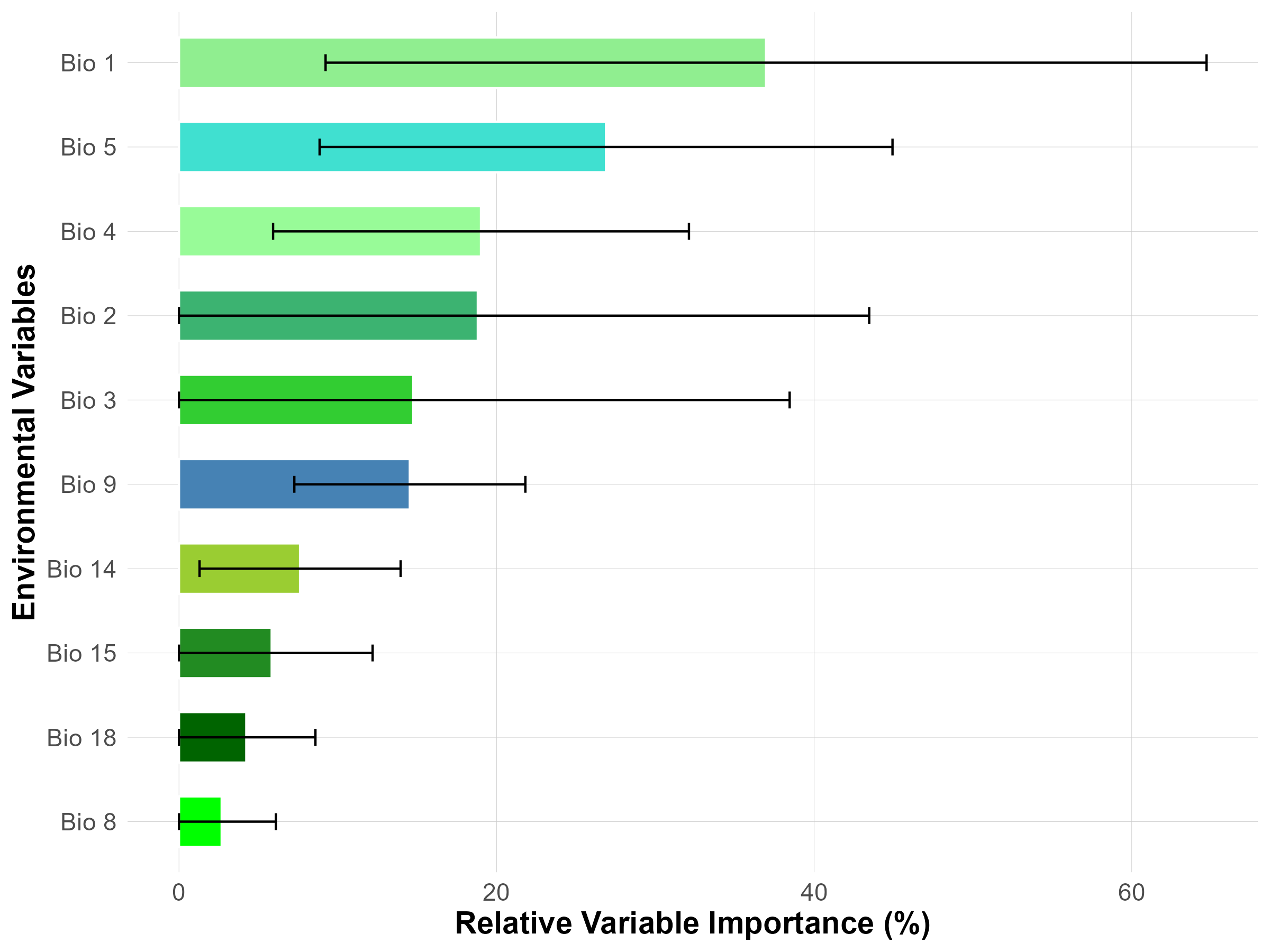

Supplement: Supplementary file 1 [file biology-14-00047-s001.zip › Figure S2.tif]
